# Serrated polyposis syndrome with multiple inverted lesions in the colon: Case report and elucidation of morphogenetic mechanism

**DOI:** 10.1002/deo2.13

**Published:** 2021-07-05

**Authors:** Chihiro Yoshikawa, Fumikazu Koyama, Kohei Morita, Hiroyuki Kuge, Takayuki Nakamoto, Shinsaku Obara, Yosuke Iwasa, Tomohiro Kunishige, Chiho Ohbayashi, Masayuki Sho

**Affiliations:** ^1^ Department of Surgery Nara Medical University Nara Japan; ^2^ Division of Endoscopy Nara Medical University Hospital Nara Japan; ^3^ Department of Diagnostic Pathology Nara Medical University Nara Japan

**Keywords:** depressed surface, endophytic growth, frustoconical crypt, inverted sessile serrated lesions, serrated polyposis syndrome

## Abstract

A 70‐year‐old man underwent surveillance colonoscopy following surgery for occlusive sigmoid colon cancer. The procedure revealed nine sessile serrated lesions (SSLs), including three inverted lesions. Endoscopic and surgical resections were performed. All nine lesions were confirmed pathologically as SSL, and the patient was diagnosed with serrated polyposis syndrome (SPS). Three inverted SSLs (iSSLs) showed endophytic growth without epithelial misplacement. Crypt analysis revealed that iSSL crypts were wider at the bottom than the opening, roughly resembling a frustoconical shape. Our results suggest that a horizontal arrangement of frustoconical crypts leads to hemispherical deformation of the muscularis mucosa, forming an inverted shape. This is the first report to reveal the morphogenesis of iSSLs from the shape of the crypt.

## INTRODUCTION

Serrated polyps can be divided into three subtypes: hyperplastic polyps, sessile serrated lesions (SSLs), and traditional serrated adenomas. SSLs have been proposed as early precursor lesions in the serrated neoplasia pathway. Two criteria are in use for a diagnosis of serrated polyposis syndrome (SPS): more than five serrated polyps proximal to the sigmoid colon, with two measuring ≥10 mm in diameter (World Health Organization [WHO] criteria I); and ≥20 serrated polyps of any size or location (WHO criteria II).^1^ We encountered a case of SPS with multiple inverted SSLs (iSSLs) in which we performed crypt analysis of SSLs and normal mucosa to elucidate the underlying morphogenetic mechanism of iSSLs. This study was conducted according to the principles of the Declaration of Helsinki. Written informed consent was obtained from the patient for the publication of this case report and any accompanying images.

## CASE REPORT

**FIGURE 1:**
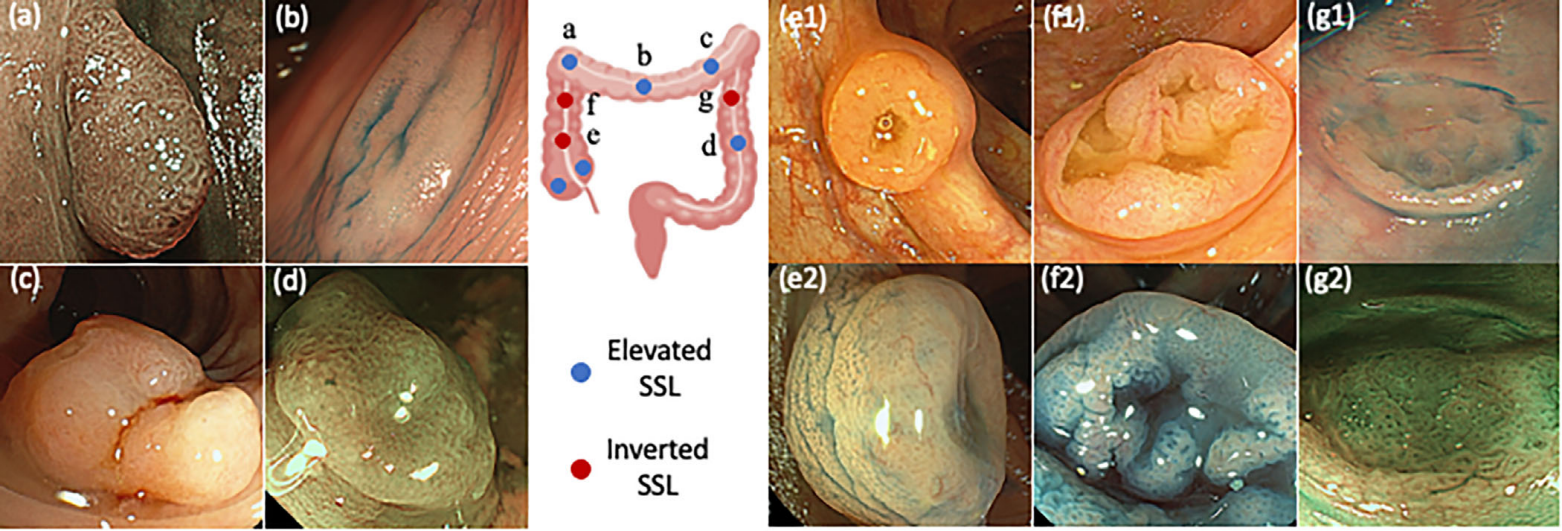
Distribution of nine SSLs in the colon and their colonoscopic appearances. Colonoscopic images are shown for all SSLs except two eSSLs in the ascending colon, which were not detected preoperatively and therefore no colonoscopic images are available. Endoscopic images of eSSLs (a–d) and iSSLs (e–g) are shown. White light appearance (e1) and indigo‐carmine dye splaying image (e2) of an iSSL 10 mm in diameter in the ascending. White light appearance (f1) and indigo‐carmine dye splaying image (f2) of an iSSL 18 mm in diameter in the ascending colon. Indigo carmine dye splaying image (g1) and blue laser imaging (g2) of iSSL in the descending colon

A 70‐year‐old Japanese man underwent colonoscopy 9 months after surgery for Stage IIA occlusive sigmoid colon cancer (T3, N0, M0) (TNM 8th edition) (type 2, 55 × 65 mm; moderately differentiated adenocarcinoma). Colonoscopy revealed seven polyps (two in the ascending, three in the transverse, and two in the descending colon) (Figure 1). Four polyps had an elevated surface (eSSL) (Figure 1a–d) and three had an inverted shape (iSSLs, Figure 1e–g). Magnification endoscopy with blue laser imaging (BLI) and narrow band imaging (NBI) revealed that seven polyps had partially dilated vessels without meshed capillary pattern (type I capillary pattern according to Sano's classification^2^). Chromoendoscopy with indigo carmine showed that seven polyps had the type II‐open or type II‐long like pit pattern proposed by Kimura.^3^ Based on these endoscopic findings, all seven polyps were considered SSLs. Of the three iSSLs, two in the ascending colon had clear depressions, with diameters of 10 mm (Figure 1e) and 18 mm (Figure 1f), whereas the iSSL in the descending colon had a shallow depression 10 mm in diameter (Figure 1g). In particular, the deep depressions of the two iSSLs in the ascending colon raised concerns about possible association with diverticula or possible undiagnosed submucosal cancer. After adequate informed consent, a laparoscopic ileocecal resection was performed as the treatment for the two iSSLs. Two other elevated polyps (no colonoscopic images) without a preoperative diagnosis were resected simultaneously. Four eSSLs (Figure 1a–d) and one iSSL (Figure 1g) in the descending colon were removed by endoscopic mucosal resection. In total, nine polyps were resected endoscopically or surgically.

**FIGURE 2:**
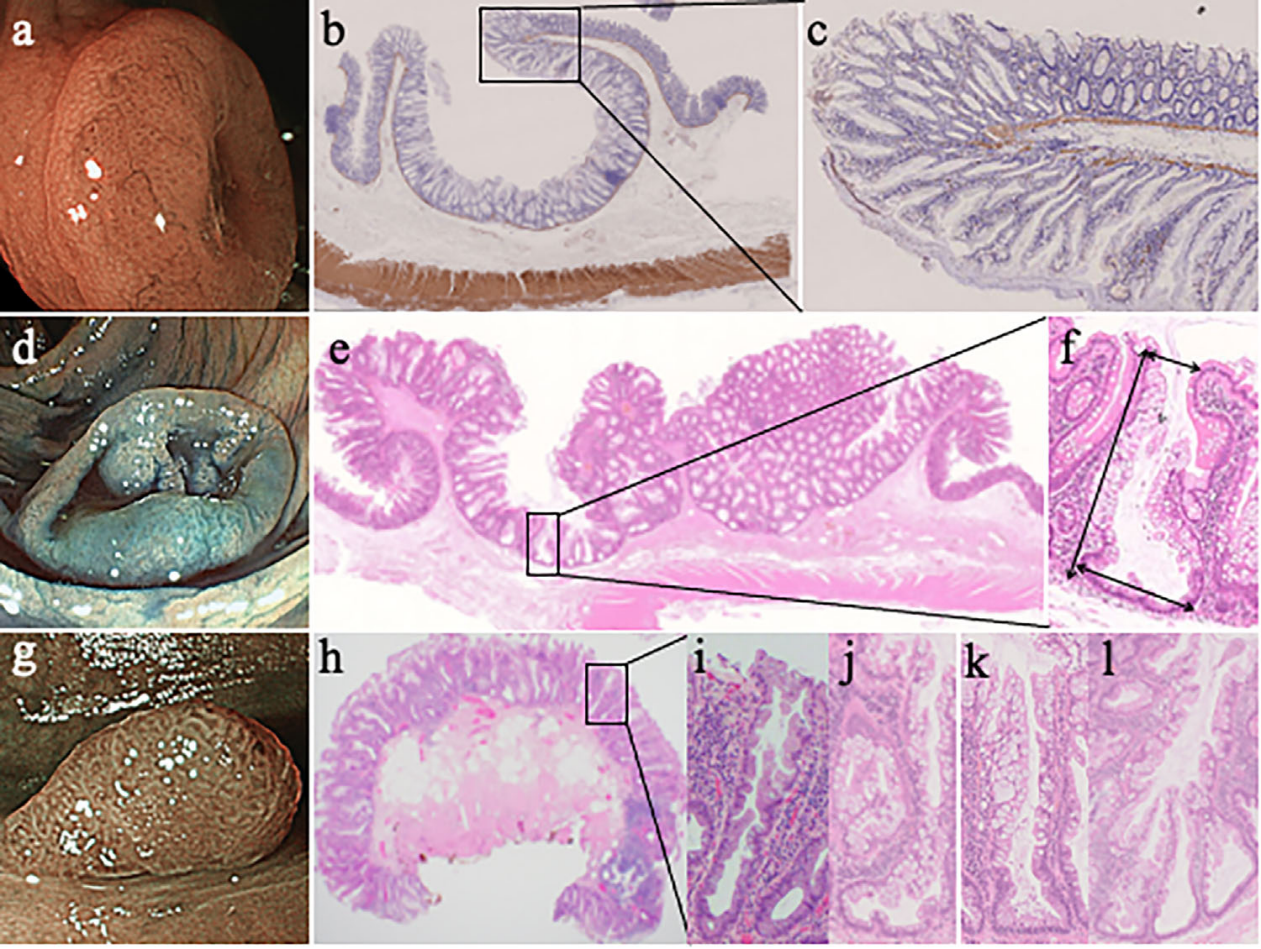
Histological images of iSSLs and eSSLs. (a) Blue laser imaging of an iSSL 10 mm in diameter in the ascending colon. (b) Histopathologic image of this iSSL. The proper muscular layer is preserved. The depressed portions comprise serrated crypts with inverted growth, and the marginal elevations comprise normal crypts (Desmin stain, 2 ×). (c) High‐magnification image of a bend section. The muscularis mucosa is bent to form circumferential ridges at the boundary between the serrated crypts and the surrounding normal crypts (Desmin stain, 2 ×). (d) Indigo‐carmine dye splaying image of an iSSL 18 mm in diameter, with minute elevations seen inside the central wide depression in the ascending colon. (e) The lesions have depressed areas with endophytic growth. Crypt misplacement is not observed (H&E stain, 2 ×). (f) Enlarged image of a crypt from a depressed part of the iSSL. The bottom of the crypt was wider than the opening, and crypt length was greater than that of normal crypts. We measured arrows as crypt length and the widths of crypt openings and bottoms. (g) Colonoscopic image of an eSSL. (h) Histopathologic image of eSSL (H&E stain, 2 ×). (i) Enlarged image of a crypt composed of eSSLs. The widths of the bottom and the opening are the same. (j) Histopathologic image of L‐shaped crypt in the 18‐mm diameter iSSL. (k) Histopathologic image of inverted T‐shaped crypt in the 18‐mm diameter iSSL. (l) Histopathologic image of irregularly branching crypts in the 18‐mm diameter iSSL.

Pathologically, all nine polyps were SSLs, and the patient was diagnosed with SPS according to the WHO diagnostic criteria I.^1^ Muscularis propria preserved in the iSSLs with the deeply depressed surface (Figure 2b and e) were neither associated with colonic diverticula nor with submucosal invasive cancer. The crypts forming the lesions were serrated with endophytic growth, without misplacement to the submucosa in the depressed portions, and with normal crypts in the marginal elevations (Figure 2b and e). Immunostaining for desmin showed that the muscularis mucosa was bent to form circumferential ridges at the boundary between the serrated crypts and the normal crypts surrounding them (Figure 2c).

We investigated the difference in crypt morphology between SSLs and normal mucosa. From digital photographs of hematoxylin and eosin‐stained sections including six eSSLs, three iSSLs, and nine nearby normal mucosa, we extracted continuously observable crypts from the opening to the bottom. Then, the length of the crypt, the width of the opening and the bottom of the crypt were measured (Figure 2f, arrow). A crypt with a bottom 2.5 times wider than that of a normal crypt was defined as a dilated crypt, and the proportions of dilated crypts in eSSLs and iSSLs were examined.

**FIGURE 3:**
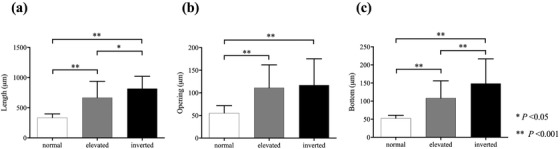
The results of crypt analysis of normal mucosa, eSSLs, and iSSLs. Statistical differences in crypt length (a), opening (b), and bottom (c) between normal mucosa, eSSLs, and iSSLs were analyzed with one‐way ANOVA and Tukey's HSD post hoc test. (a) Mean crypt length in normal mucosa, eSSLs, and iSSLs was 335.7 ± 62.0, 668.7 ± 269.4, and 817.7 ± 204.6, respectively. (b) Mean width of the crypt opening in normal mucosa, eSSLs, and iSSLs was 55.3 ± 16.5, 111.3 ± 50.5, and 117.0 ± 58.4, respectively. (c) Mean width of the crypt bottom in normal mucosa, eSSLs, and iSSLs was 52.6 ± 8.2, 108.3 ± 47.7, and 148.6 ± 68.2, respectively.

Crypt length was significantly greater in eSSLs than in normal mucosa (*p* < 0.001), and significantly greater in iSSLs than in normal mucosa (*p* < 0.001) and eSSLs (*p* < 0.05) (Figure 3a). Width of the crypt opening was significantly greater in eSSLs and iSSLs than in normal mucosa (*p* < 0.001); however, there was no significant difference between eSSLs and iSSLs in terms of width of crypt opening (Figure 3b). Width of the crypt bottom was significantly greater in eSSLs than in normal mucosa (*p* < 0.001), and was significantly greater in iSSLs than in eSSLs (*p* < 0.001) (Figure 3c). The proportion of dilated crypts in iSSL (60.7%) was significantly higher than that in eSSL (23.1%) (*p* < 0.05).

## DISCUSSION

SPS, formerly termed hyperplastic polyposis syndrome, is a common but underrecognized colorectal polyposis syndrome that is associated with increased risk of colorectal cancer. The present case had SPS with multiple iSSLs. Reports of SPS with iSSLs are limited to one case each of SPS^4^ and hyperplastic polyposis syndrome.^5^ The characteristic endoscopic finding of SSLs is a slightly elevated pale polyp covered by a thin "mucus cap." In clinical practice, iSSL is often encountered. The frequency of iSSL in SSL has been reported to be 10.4–35.6%,^6^, ^7^ but the exact frequency remains unclear.

Regarding the morphogenesis of serrated polyps with an inverted shape, Sobin first reported inverted hyperplastic polyps and advocated a “passive congestion” theory that favored downward proliferation, termed endophytic growth.^8^ Stepherd proposed the mild traction theory, in which epithelial misplacement occurs via pre‐existing defects in the muscularis mucosae.^5^ Following the publication of that report, epithelial misplacement has often been referred to as a histologic feature of inverted serrated polyps.^9^ However, epithelial misplacement was not observed in nine of 10 iSSLs reported by Sobin,^8^ or in the three iSSLs in the present case. In addition, the three iSSLs did not have dysplasia. Therefore, epithelial misplacement is unlikely to be an essential morphogenetic mechanism of iSSLs. The characteristic histologic findings of SSLs include serration extending to the base of the crypts, dilated and inverted T‐ or boot‐shaped crypts, and crypt branching.^10^ A recent report found that inverted shape of serrated polyps was specific to SSLs.^6^ This finding suggests that the crypt morphology of SSLs may affect the gross appearance.

**FIGURE 4:**
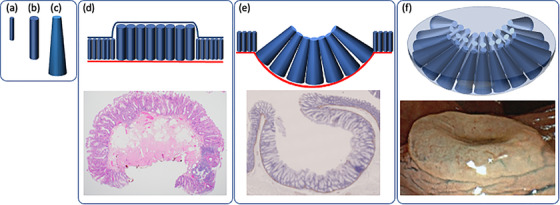
Graphic representation of crypt types and the morphogenetic mechanism. (a) Normal, (b) eSSL, and (c) iSSL crypts. (d) Image showing crypt arrangement in an eSSL. The elevated portions consist of large columnar crypts. (e) Image showing crypt arrangement in an iSSL. The depressed portions consist of frustoconical crypts, and the marginal elevations contain columnar shaped crypts. The red line indicates the muscularis mucosa, which bends to form a circumferential ridge at the boundary between the columnar crypts and surrounding normal crypts. (f) 3D image of an iSSL. The frustoconical crypts grow in a circular arrangement, compressing the muscularis mucosa convexly toward the submucosal layer, to form a hemispherical surface.

To clarify whether crypt morphology affects the gross appearance of SSLs, we performed crypt analysis in the present case. The results showed that SSL had a significantly longer crypt length and a significantly wider crypt opening and bottom compared to the normal crypt (Figure 3). There was no significant difference in the width of the crypt opening between eSSL and iSSL (Figure 3a), but the bottom of the crypt was significantly wider in iSSL than in eSSL (Figure 3c). The shape of the individual crypts included a variety of complex shapes, including inverted T‐shaped and/or L‐shaped crypts (Figure 2j and k) and irregularly branched crypts (Figure 2l). On average, the crypt shapes of normal mucosa, eSSL, and iSSL could be approximated to “columnar” (Figure 4a), “larger columnar” (Figures 2i and 4b), and “larger frustoconical,” respectively. It is reasonable that eSSLs would have an elevated surface, as they have larger columnar crypts compared to normal mucosa. However, the surfaces of iSSLs were depressed, despite having larger crypts than the surrounding normal mucosa. On the basis of these results, we performed a simulation of the arrangement of columnar and frustoconical crypt models (Figure 4). When frustoconical crypts were arranged in a monolayer on the muscularis mucosa, a hemispherical depression was formed at their confluence due to the difference in area between the top and bottom. When frustoconical crypts continue to grow in a limited space, the muscularis mucosa is compressed in a convex manner toward the submucosal layer of the lesion and is refracted to form circumferential ridges at the boundary with columnar crypts. Accordingly, we consider that proliferation of frustoconical crypts is a morphogenetic mechanism of iSSLs. If there is only a small difference in area between the top and bottom of frustoconical crypts, the depression will be shallow (Figure 1g). In large iSSLs, refraction of the muscularis mucosa occurs at multiple locations, resulting in a complex macroscopic appearance (Figures 1f and 2e). Excessive stretching and refraction of the muscularis mucosa are considered to cause crypt misplacement, but iSSLs can form even without crypt misplacement.

It is a limitation of this study that crypt analyses were performed in only one patient with SPS. However, it has the advantage of avoiding the effects of interindividual disparity because we examined multiple lesions in the same patient. The number or frequency of frustoconical crypt might associated with morphogenetic mechanism of iSSLs, and further research will be necessary. This is the first case report worldwide to elucidate the mechanism of endophytic growth of iSSLs from the crypt morphology.

## CONFLICT OF INTEREST

The authors declare no conflicts of interest.

## FUNDING INFORMATION

None.
